# Suppression of Inflammation in Adipocyte-Macrophage Coculture by Passion Fruit Seed Extract: Insights into the p38 and NF-ҡB Pathway

**DOI:** 10.1155/2024/7990333

**Published:** 2024-03-09

**Authors:** Sukanya Chusongdam, Wanwipha Woonnoi, Furoida Moolsup, Chakkapat Aenglong, Pennapa Chonpathompikunlert, Supita Tanasawet, Jirawat Saetan, Wanida Sukketsiri

**Affiliations:** ^1^Division of Health and Applied Sciences, Faculty of Science, Prince of Songkla University, Hat Yai, Songkhla 90110, Thailand; ^2^Laboratory Animal Service Center, Faculty of Science, Prince of Songkla University, Songkhla 90110, Thailand; ^3^Biodiversity Research Centre, Thailand Institute of Scientific and Technological Research (TISTR), Pathumthani 12120, Thailand

## Abstract

Obesity, which is characterized by chronic low-grade inflammation, involves the infiltration of immune cells into adipose tissue, leading to the secretion of inflammatory cytokines and subsequent inflammation. Therefore, the aim of this study was to examine the potential of passion fruit seed extract (PSEE) in mitigating lipopolysaccharide (LPS)-induced inflammation in a coculture system comprising macrophages and adipocytes. PSEE demonstrated significant reductions in reactive oxygen species (ROS) and nitric oxide (NO) levels, primarily achieved through the downregulation of inducible nitric oxide synthase (iNOS) protein expression in LPS-induced adipocyte-macrophage cocultures. Furthermore, PSEE effectively suppressed the secretion of TNF-*α* and IL-1*β* by attenuating the gene expression of these cytokines, as well as other inflammation-related genes such as MMP-2, IL-6, and MCP-1. Notably, PSEE exhibited potent inhibitory effects on the p38 and NF-*κ*B signaling pathways, thus alleviating inflammation in the LPS-induced adipocyte-macrophage cocultures. Additionally, PSEE led to a decrease in the expression of ACC, HSL, and FaSN, while aP2 and ATGL showed increased expression in LPS-induced cocultured macrophages and adipocytes. These findings suggest that passion fruit seed extract effectively combats inflammation by suppressing the p38 and NF-*κ*B signaling pathways, resulting in reduced levels of proinflammatory cytokines, NO, and ROS production.

## 1. Introduction

Obesity, characterized by the excessive accumulation of fat, is a chronic low-grade systemic inflammation that significantly increases the risk of various metabolic diseases, including cardiovascular disease, hypertension, type II diabetes, and other inflammation-related disorders [1]. This inflammatory state is primarily attributed to chronic inflammation within adipose tissue, which involves the infiltration of macrophages and other inflammatory cells, accompanied by the release of proinflammatory cytokines, all of which are closely associated with obesity [2]. The presence of macrophages in adipose tissue promotes the secretion of proinflammatory cytokines such as tumor necrosis factor alpha (TNF-*α*), monocyte chemotactic protein-1 (MCP-1), and interleukin 6 (IL-6), leading to the initiation of an inflammatory response and the development of insulin resistance within adipose tissue [3]. Consequently, reducing macrophage-induced inflammation in adipose tissue could potentially mitigate the progression of obesity-related diseases.

The coculture of adipocytes and macrophages is essential for studying various physiological and pathological processes [4]. The interaction between these two cell types plays a significant role in the development of inflammation and metabolic disorders [4–6]. In obesity, the cross-talk between adipocytes and macrophages can lead to the secretion of proinflammatory factors, contributing to chronic low-grade inflammation. This interaction has been studied using in vitro coculture systems, which mimic the in vivo environment and help in understanding the complex cellular interactions and signaling pathways involved [7, 8]. Therefore, cocultured adipocytes and macrophages serve as a valuable tool for investigating the pathophysiology of various metabolic conditions and for the development of potential therapeutic interventions.

Various treatments for obesity, such as orlistat and lorcaserin, have been associated with certain limitations, including side effects. Orlistat can lead to side effects such as oily stools and increased defecation. Lorcaserin is associated with side effects such as serotonin syndrome [9]. *Passiflora edulis* Sims f. flavicarpa, a variety of passion fruit known for its yellow or sour flavor, has been extensively studied for its diverse range of biological activities in both *in vitro* and *in vivo* settings. These properties include antioxidant, anti-inflammatory, antimicrobial, antihypertensive, hepatoprotective, and antidiabetic activities [10–12]. In a previous study, our study reported that the seed extract of *P. edulis* is particularly rich in total phenolics, flavonoids, carotenoids, and stilbenes, with a notable concentration of piceatannol [12]. Moreover, passion fruit seed extract has demonstrated remarkable biological activity, including antioxidant and anti-inflammatory properties, inhibition of pancreatic lipase and cholesterol esterase, as well as vasorelaxation in rat aortic rings [12, 13]. Despite the well-known anti-inflammatory effects of piceatannol, no previous studies have explored these effects specifically in relation to passion fruit seed extract. Therefore, the objective of this study was to investigate the inhibitory effects of passion fruit seed ethanolic extract (PSEE) on lipopolysaccharide (LPS)-induced inflammatory responses in a coculture model consisting of 3T3-L1 adipocytes and RAW264.7 macrophages.

## 2. Materials and Methods

### 2.1. Preparation of Passion Fruit Seed Extract

To obtain the passion fruit seed ethanolic extract (PSEE), the seeds were first dried and then powdered. The powdered seeds were mixed with 70% ethanol in a ratio of 1 : 10. The mixture was allowed to extract at room temperature for 72 h. Subsequently, the extract solution was filtered using Whatman® paper No. 1. The solvent was then evaporated using an evaporator (Buchi, Switzerland), and the remaining extract was dried using a freeze dryer. The resulting extract sample was stored in amber glass bottles at −20°C for further analysis. The phytochemical analysis of the extract was previously reported in our study [12].

### 2.2. Cell Culture

In this study, murine 3T3-L1 preadipocytes (ATCC, USA) and murine macrophages RAW 264.7 (ATCC, USA) cells were cultured separately using different culture media. The 3T3-L1 cells were cultured in Dulbecco's Modified Eagle Medium (DMEM; Gibco, USA) high glucose (with a glucose content of 4.5 g/L). On the other hand, the RAW 264.7 cells were cultured in DMEM low glucose (with a glucose content of 1.0 g/L; Gibco, USA). Both culture media were supplemented with 10% heat-inactivated fetal bovine serum (Gibco, USA), 1% penicillin-streptomycin, and 1% L-glutamine (Gibco, USA). Both cell lines were incubated at a temperature of 37°C, in a humidified atmosphere with 5% CO_2_.

### 2.3. Adipocyte Differentiation and Coculture with Macrophages

The 3T3-L1 preadipocytes were induced to differentiate into mature adipocytes by treating them with the differentiation medium comprising DMEM high glucose, supplemented with 0.25 *μ*M dexamethasone (Sigma, USA), 0.5 mM 3-isobutyl-1-methylxanthine (IBMX; Sigma, USA), and 1 *μ*g/mL insulin (Sigma, USA) [14]. Additionally, the adipocytes were treated with various concentrations of PSEE (10, 50, and 100 *μ*g/mL) for a duration of 12 days to determine the effect of PSEE on adipogenesis. After the 12-day differentiation period, RAW264.7 macrophages were cocultured with the 3T3-L1 mature adipocytes in a ratio of 4 : 1 (3T3-L1 mature adipocytes: macrophages) according to previous studies with some modification [15–17]. The coculture was incubated for 4 h to allow the macrophages to adhere to the mature adipocytes. Subsequently, 1 *μ*g/mL of lipopolysaccharide (LPS) derived from *Escherichia coli* (Sigma, USA) was added to the coculture and incubated for 24 h.

### 2.4. Cytotoxicity Test

To assess cytotoxicity in the cocultured cells, the 3-(4,5-dimethylthiazole-2-yl)-2,5-diphenyltetrazolium bromide (MTT) assay was performed. Following the treatment protocol, the cocultured cells were incubated with MTT solution at a concentration of 500 *μ*g/mL for a duration of 2 h at 37°C. After the incubation period, the absorbance of the cells was assessed at 570 nm using a microplate reader (SPECTROstar Nano, BGM Labtech, Germany).

### 2.5. Intracellular Reactive Oxygen Species (ROS) Production

To analyze the production of reactive oxygen species (ROS) in the cocultured cells, the 2′-7′-dichlorofluorescin diacetate (DCFH_2_-DA) assay was conducted. After 24 h of LPS treatment, the cocultured medium was removed, and the cells were washed with PBS. Subsequently, 100 *μ*L of 50 µM DCFH_2_-DA (Sigma, USA) was added to the cells and incubated for 1 h at a temperature of 37°C. After the incubation period, the DCFH_2_-DA solution was removed, and the cells were washed with PBS. The fluorescence intensity of the cells was then measured using a fluorescence microplate reader (Bio-tex, USA) at an excitation wavelength of 485 nm and an emission wavelength of 535 nm.

### 2.6. Nitric Oxide Assay

The level of nitric oxide (NO) was determined using the Griess assay. For each treatment group, 100 *μ*L of the cocultured medium was combined with 100 *μ*L of Griess reagent (Sigma, USA). The resulting mixture was then incubated for a duration of 10 min. After the incubation period, the NO level in the samples was detected at 540 nm using a microplate reader. Sodium nitrate was used as a standard for comparison in order to quantify the nitric oxide levels in the cocultured samples.

### 2.7. Determination of TNF-*α* and IL-1*β* Level

The TNF-*α* and IL-1*β* levels were determined using an ELISA assay (Merck Millipore, Burlington, Massachusetts, USA). To perform the assay, 100 *μ*L of the cocultured medium was added to each well of a 96-well ELISA plate. The plate was then incubated for 2.5 h at room temperature with gentle shaking. After the incubation, the plate was rinsed four times with 1X Wash Solution. Then, the prepared Detection Antibody (100 *μ*L) was applied to each well and incubated at room temperature for 60 min with gentle shaking. Following this, the prepared streptavidin solution (100 *μ*L) was applied to each well and incubated at room temperature with gentle shaking for 45 min. Next, the TMB One-Step Substrate Reagent (100 *μ*L) was incubated for 30 min. Finally, the reaction was stopped by adding the stop solution and measured at 450 nm using a microplate reader.

### 2.8. Real Time Reverse Transcriptase-Polymerase Chain Reaction (qRT-PCR)

Total RNA was extracted from the cocultured 3T3-L1 and RAW264.7 cells using the TRIzol reagent. The concentration of the extracted RNA was measured using a Bio spectrometer (Eppendorf, Hamburg, Germany). The extracted RNA was then converted into complementary DNA (cDNA) using a cDNA synthesis kit (Invitrogen, Waltham, Massachusetts, USA). The cDNA template was mixed with the SensiFAST SYBR NO-ROX kit (Invitrogen™, USA) and quantified using a real-time PCR detection system (Model FQD-96A, Bioer, China). The quantification was performed using SYBR green dye to examine the mRNA expression levels of various genes, including ACC, aP2, FaSN, ATGL, HSL, LPL, leptin, resistin, Glut4, IsnR, adipoQ, and adiponectin receptors (adipoQ-R1 and adipoQ-R2). Additionally, the expressions of TNF-*α*, IL-1*β*, IL-6, MCP-1, MMP-2, and MMP-9 were also evaluated. The housekeeping gene *β*-actin was used as a reference gene for normalization. The gene expression analysis was performed using the 2^−ΔΔCt^ method, which allows for the comparison of relative gene expression levels between different samples.

### 2.9. Western Blot Analysis

The cocultured cells were washed with PBS and subsequently lysed using RIPA buffer. The lysis process was conducted for 30 min at a temperature of 4°C. Afterward, the lysed cells were disrupted using a homogenizer and the resulting mixture was centrifuged at 14,000 rpm for 20 min. The total protein concentration in the lysate was measured using Bradford's method. Then, 75 *μ*g of protein was loaded onto 10% sodium dodecyl sulfate-polyacrylamide gel electrophoresis and transferred onto PDVF membranes. The membranes were blocked with 5% skim milk for 2 h and washed with 1X TBST for 1 h. The membranes were then incubated with primary antibodies to ERK1/2 (Abcam, Cambridge, UK), pERK1/2 (Santa Cruz Biotechnology, CA, USA), p-p38 (Santa Cruz Biotechnology), p38 (Santa Cruz Biotechnology), nuclear factor-*κ*B (NF-*κ*B; Santa Cruz Biotechnology), and inducible nitric oxide synthase (iNOS; Santa Cruz Biotechnology) overnight at 4°C. The membranes were then incubated with horseradish peroxidase-conjugated secondary antibody for 2 h at room temperature. The band of proteins was observed by the ECL detection reagent, followed by exposure to X-ray hyperfilm, and the relative band density was measured by normalizing with *β*-actin using ImageJ software (National Institutes of Health, Bethesda, MD, USA).

### 2.10. Statistical Analysis

All data were reported as the mean ± standard error of the mean (SEM). Statistical comparisons were conducted using one-way analysis of variance (ANOVA), followed by a post-hoc LSD (Least Significant Difference) test using SPSS software. A *p* value less than 0.05 was considered statistically significant to indicate meaningful differences between the groups.

## 3. Results

### 3.1. Effects of PSEE against ROS and NO Production on LPS-Induced Inflammation in Cocultured Adipocytes and Macrophages

**Figure 1:**
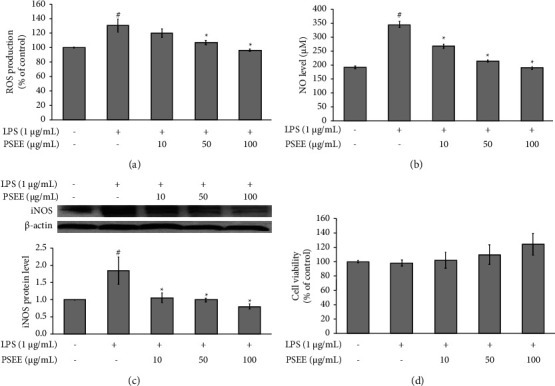
Inhibition of reactive oxygen species (ROS) and nitric oxide (NO) production by PSEE in lipopolysaccharide (LPS)-induced inflammation in cocultured adipocytes and macrophages. The 3T3-L1 preadipocytes were induced to undergo differentiation into mature adipocytes and subsequently treated with PSEE (10, 50, and 100 *μ*g/mL) for a duration of 12 days. Following the 12-day differentiation phase, coculture was established with RAW264.7 macrophages and mature 3T3-L1 adipocytes. Inflammation was induced by the addition of 1 *μ*g/mL of LPS for a 24 h period. (a) Intracellular ROS production. (b) NO level. (c) Inducible nitric oxide synthase (iNOS) protein expression. (d) Cytotoxicity. All values are presented as the mean ± SEM from four independent experiments (*n* = 4). Statistical significance was determined using LSD *post-hoc* analysis, with significance accepted when *p* < 0.05 (# compared to the control group and ^*∗*^compared to the LPS group).

According to Figure 1(a), the production of ROS in the LPS group was significantly higher compared to the control group (*p* ≤ 0.05). Interestingly, treatment with PSEE at concentrations of 50 and 100 *μ*g/mL resulted in a significant decrease in ROS production compared to the LPS group (Figure 1(a); *p* ≤ 0.05). As shown in Figures 1(b) and 1(c), the highest level of NO and iNOS protein expression was found in the LPS group compared to the control group (*p* ≤ 0.05). However, treatment with all concentrations of PSEE resulted in significantly lower NO levels and iNOS protein expression compared to the LPS group (*p* ≤ 0.05) (Figures 1(b) and 1(c)). Lastly, the effect of long-term treatment with PSEE on cell viability in cocultured adipocytes and macrophages was investigated (Figure 1(d)). The results indicated that neither LPS nor the different concentrations of PSEE (10, 50, and 100 *μ*g/mL) had a significant effect on cell viability, suggesting that PSEE treatment did not exhibit cytotoxicity in the cocultured cells (Figure 1(d)). Overall, these results demonstrated the potential of PSEE in reducing LPS-induced ROS and NO secretion without causing cytotoxicity in cocultured adipocytes and macrophages.

### 3.2. Effects of PSEE against the Secretion and Expression of TNF-*α* and IL-1*β* on LPS-Induced Inflammation in Cocultured Adipocytes and Macrophages

**Figure 2:**
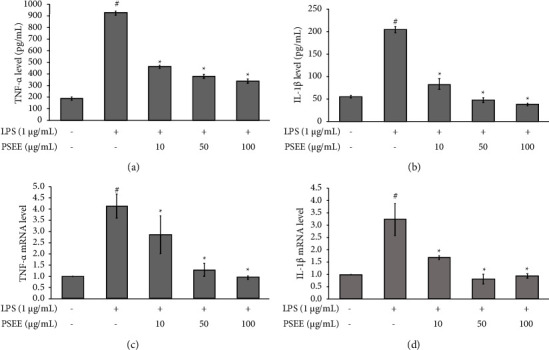
Inhibition of TNF-*α* and IL-1*β* secretion and gene expression by PSEE in lipopolysaccharide (LPS)-induced inflammation in cocultured adipocytes and macrophages. The 3T3-L1 preadipocytes were induced to differentiate into mature adipocytes and treated with PSEE (10, 50, and 100 *μ*g/mL) for a duration of 12 days. Following the 12-day differentiation phase, coculture was established with RAW264.7 macrophages and mature 3T3-L1 adipocytes. Inflammation was induced by the addition of 1 *μ*g/mL of LPS for a 24 h period. (a) Secretion of TNF-*α*. (b) Secretion of IL-1*β*. (c) Gene expression of TNF-*α*. (d) Gene expression of IL-1*β*. All values are expressed as the mean ± SEM from four independent experiments (*n* = 4). ^*∗*^Significantly different from the LPS group; #significantly different from the control group. Statistical significance was measured using the LSD *post-hoc* test (*p* < 0.05).

Tumor necrosis factor-alpha (TNF-*α*) and interleukin-1beta (IL-1*β*) are proinflammatory cytokines that play crucial roles in regulating inflammatory responses, cell differentiation, proliferation, and apoptosis. In this study, we aimed to investigate whether PSEE could downregulate the secretion and expression of TNF-*α* and IL-1*β* in cocultured 3T3-L1 adipocytes and macrophages induced inflammation by LPS. The release of TNF-*α* and IL-1*β* was measured using ELISA, and the results are shown in Figures 2(a) and 2(b), respectively. Treatment with all concentrations of PSEE significantly attenuated the release of TNF-*α* and IL-1*β* compared to the LPS group (Figures 2(a) and 2(b); *p* ≤ 0.05). Furthermore, the mRNA expression levels of TNF-*α* and IL-1*β* were evaluated, as shown in Figures 2(c) and 2(d), respectively. Treatment with PSEE (10, 50, and 100 *μ*g/mL) resulted in a significant downregulate in the mRNA expression of TNF-*α* and IL-1*β* compared to the LPS group (Figures 2(c) and 2(d); *p* ≤ 0.05). These findings indicated that PSEE has the ability to inhibit inflammation in cocultured adipocytes and macrophages by suppressing the expression and release of proinflammatory cytokines, specifically TNF-*α* and IL-1*β*.

### 3.3. Effects of PSEE against the Expression of Cytokines and Adipokines on LPS-Induced Inflammation in Cocultured Adipocytes and Macrophages

**Figure 3:**
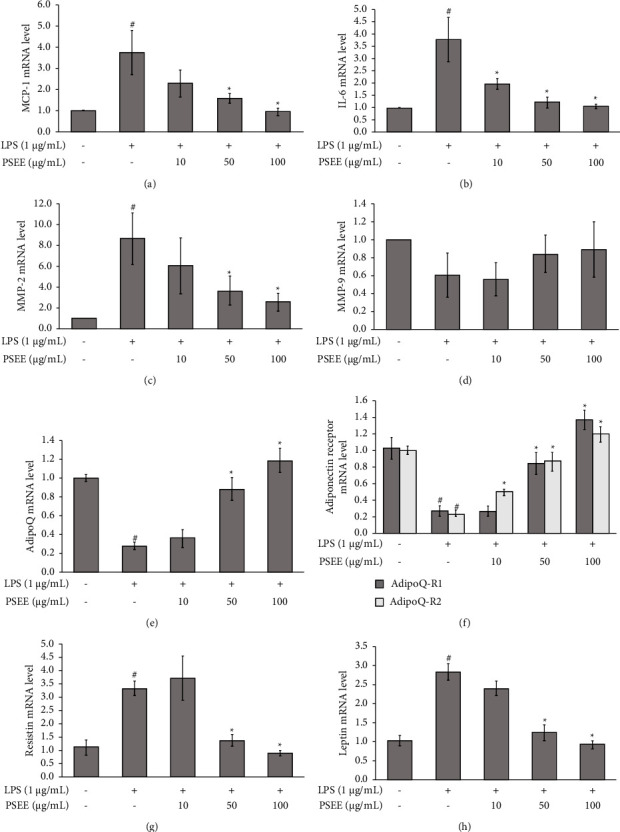
Inhibition of cytokine and adipokine gene expression by PSEE in lipopolysaccharide (LPS)-induced inflammation in cocultured adipocytes and macrophages. The 3T3-L1 preadipocytes were induced to differentiate into mature adipocytes and treated with PSEE (10, 50, and 100 *μ*g/mL) for a duration of 12 days. Following the 12-day differentiation phase, coculture was established with RAW264.7 macrophages and mature 3T3-L1 adipocytes. Inflammation was induced by the addition of 1 *μ*g/mL of LPS for a 24-h period. (a) MCP-1 mRNA expression. (b) IL-6 mRNA expression. (c) MMP-2 mRNA expression. (d) MMP-9 mRNA expression. (e) adipoQ mRNA expression. (f) adipoQ-R1 and adipoQ-R2 mRNA expression. (g) Leptin mRNA expression. (h) Resistin mRNA expression. All values are expressed as the mean ± SEM from four independent experiments (*n* = 4). ^*∗*^Significantly different from the LPS group; #significantly different from the control group. Statistical significance was measured using the LSD *post-hoc* test (*p* < 0.05).

In this study, we investigated the gene expression levels of matrix metallopeptidase (MMP)-2, MMP-9, interleukin-6 (IL-6), monocyte chemotactic protein-1 (MCP-1), adiponectin, adiponectin receptors, leptin, and resistin in the cocultured adipocytes and macrophages. As shown in Figures 3(a)–3(c), the highest levels of MCP-1, IL-6, and MMP-2 gene expression were observed in the LPS group (*p* ≤ 0.05). Treatment with PSEE at concentrations of 50 and 100 *μ*g/mL significantly downregulated the expression levels of MCP-1, IL-6, and MMP-2 compared to the LPS group (Figures 3(a)–3(c); *p* ≤ 0.05). However, the expression of MMP-9 was not significantly affected by the treatment with PSEE (Figure 3(d)). In addition, treatment with PSEE at concentrations of 50 and 100 *μ*g/mL significantly upregulated the expression levels of adiponectin and adiponectin receptors (adipoQ-R1 and adipoQ-R2) (Figures 3(e) and 3(f)). In contrast, PSEE (50 and 100 *μ*g/mL) caused a significant decrease in leptin and resistin mRNA expression in the cocultured adipocytes and macrophages (Figures 3(g) and 3(h)). These findings suggest that PSEE attenuated inflammation in the cocultured adipocytes and macrophages by suppressing the expression of cytokines and adipokines.

### 3.4. Effects of PSEE on the Expression of Lipogenic Genes and Insulin Resistance in LPS-Induced Adipocytes and Macrophages Coculture

**Figure 4:**
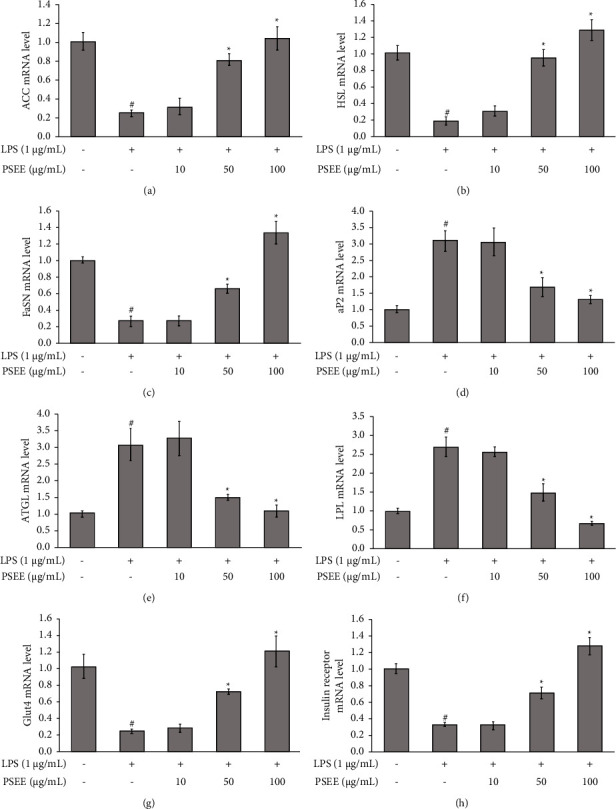
Inhibition of lipogenic genes, glucose transporter, and insulin receptor gene expression by PSEE in LPS-induced inflammation in cocultured adipocytes and macrophages. (a) ACC mRNA expression. (b) aP2 mRNA expression. (c) FaSN mRNA expression. (d) ATGL mRNA expression. (e) HSL mRNA expression. (f) LPL mRNA expression. (g) Glut4 mRNA expression. (h) Insulin receptor (IsnR) mRNA expression. All values are expressed as the mean ± SEM from four independent experiments (*n* = 4). ^*∗*^Significantly different from the LPS group; #significantly different from the control group. Statistical significance was measured using the LSD post-hoc test (*p* < 0.05).

The effects of PSEE on the expression of lipogenic genes (adipocyte fatty acid binding protein (aP2), acetyl-CoA carboxylase (ACC), fatty acid synthase (FaSN), lipoprotein lipase (LPL), hormone sensitive lipase (HSL), and adipose triglyceride lipase (ATGL)) were investigated in LPS-induced adipocytes and macrophages coculture. The results demonstrated that PSEE at the concentrations of 50 and 100 *μ*g/mL significantly increased the expression of ACC, HSL, and FaSN, while aP2, ATGL, and LPL exhibited a decrease in LPS-induced cocultured adipocytes compared to the LPS-alone condition (Figures 4(a)–4(f)). Moreover, treatment with PSEE at concentrations of 50 and 100 *μ*g/mL led to a reduction in insulin resistance in the cocultured adipocytes with macrophages, as evidenced by a significant increase in the expression of Glut4 and insulin receptor genes (Figures 4(g) and 4(h)).

### 3.5. PSEE Inhibits Inflammation through NF-*κ*B and MAPK Pathway

**Figure 5:**
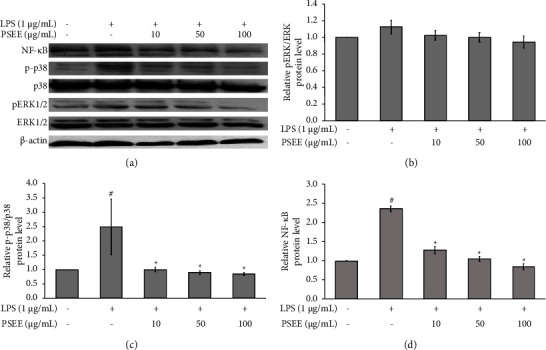
Suppression of NF-*κ*B and p38MAPK signaling pathways by PSEE in LPS-induced inflammation in cocultured adipocytes and macrophages. (a) Western blot analysis of NF-*κ*B, ERK1/2, and p38MAPK protein bands in response to PSEE treatment. (b) Relative expression of p-p38/p38 protein. (c) Relative expression of pERK/ERK protein. (d) Relative expression of NF-*κ*B protein. All values are expressed as the mean ± SEM from four independent experiments (*n* = 4). Statistical significance was determined using LSD post-hoc analysis, with significance accepted when *p* < 0.05 (# compared to the control group and ^*∗*^compared to the LPS group).

As shown in Figure 5, the NF-*κ*B and p38 expression in their active forms were significantly upregulated following LPS treatment. However, in the presence of PSEE treatment, the phosphorylation of p38 and the expression of NF-*κ*B p65 protein were significantly suppressed compared to the LPS group (Figures 5(a), 5(c) and 5(d); *p* ≤ 0.05). On the other hand, there was no significant difference in the expression levels of ERK1/2 among the experimental groups (Figures 5(a) and 5(b); *p* > 0.05). These results suggest that PSEE has the potential to significantly inhibit the activation of the inflammatory response through the NF-*κ*B and p38MAPK pathways.

## 4. Discussion

Obesity is characterized by chronic low-grade inflammation, which occurs due to the infiltration of inflammatory cells into adipose tissue [18]. This infiltration leads to the transformation of monocytes into M1 macrophages, resulting in the secretion of proinflammatory cytokines such as TNF-*α*, IL-6, IL-1*β*, and MCP-1, thereby promoting adipose tissue inflammation [19]. The interaction between adipocytes and macrophages plays a significant role in the development of chronic inflammation in adipose tissue among obese individuals [18]. The objective of this study was to investigate the inhibitory effect of PSEE on LPS-induced inflammation in a coculture of macrophages and adipocytes. Obesity is associated with increased production of free radicals, such as ROS and reactive nitrogen species, which contribute to the secretion and expression of proinflammatory cytokines [20, 21]. Elevated levels of NO are found in inflamed adipocytes and macrophages, and increased NO production has been observed in obese individuals [22]. ROS can activate signaling pathways, including NF-*κ*B and MAPK, leading to the production of proinflammatory cytokines such as TNF-*α* and IL-1*β*, as well as the expression of iNOS [23, 24]. In this study, it was observed that treatment with PSEE at concentrations of 50 and 100 *μ*g/mL significantly reduced the ROS levels in cocultured adipocytes and macrophages stimulated with LPS. Furthermore, the treatment with PSEE significantly inhibited the secretion of NO and the expression of iNOS protein in the cocultured cells stimulated with LPS. These findings align with previous research, which consistently revealed that passion fruit seed extract and its constituents, including piceatannol and resveratrol, possess the capability to diminish ROS levels, suppress NO production, and mitigate iNOS protein expression across various cell types, including keratinocytes, adipocytes, and macrophages [15, 16, 25, 26]. In our previous report, we demonstrated that passion fruit seed extract, obtained through both ethanol and water extraction methods, led to a reduction in the levels of NO in LPS-induced RAW264.7 cells [12]. Taken together, these experiments suggest that PSEE has anti-inflammatory effects in cocultured adipocytes and macrophages by inhibiting ROS and NO production through the suppression of iNOS expression.

The interaction between adipocytes and macrophages plays a significant role in chronic inflammation observed in obese individuals. Adipocytes contribute to the secretion of proinflammatory cytokines such as TNF-*α*, IL-1*β*, MMP-2, MMP-9, IL-6, and MCP-1, as well as adipokines like adiponectin, which further contribute to chronic inflammation in adipose tissue of individuals with obesity [18]. In obesity, MCP-1 is vital for macrophage infiltration into adipose tissue, where both adipocytes and macrophages release MCP-1, intensifying macrophage recruitment [8, 27]. IL-6 is predominantly produced by infiltrated adipose tissue macrophages, while elevated MMP-2 and MMP-9 levels in obesity contribute to adipose tissue remodeling, triggering proinflammatory cytokine secretion and amplifying the inflammatory response [5, 19]. In this study, the treatment of cocultured adipocytes and macrophages with PSEE resulted in a significant reduction in the release and expression of TNF-*α* and IL-1*β* compared to the LPS-only group. This finding was consistent with previous studies that demonstrated the ability of resveratrol and piceatannol to suppress the secretion of TNF-*α* and IL-1*β* [6, 16]. In addition, Li et al. [28] demonstrated that piceatannol exhibits anti-inflammatory properties by reducing inflammation through the inhibition of TNF-*α* production in cocultured adipocytes and macrophages, suppressing signaling pathways such as NF-*κ*B and JNK pathways. Furthermore, the expressions of MMP-2, IL-6, and MCP-1 genes were significantly suppressed in response to PSEE. These results were consistent with previous studies reporting that piceatannol can reduce the production of MCP-1 and IL-6 in cocultures of adipocytes and macrophages [16, 28]. Additionally, resveratrol was shown to decrease the levels of IL-6 and MCP-1 in inflamed adipocytes [29]. Moreover, *α*-tocopherol that is found in passion fruit has been shown to inhibit the expression of the IL-6 gene in cocultures of adipocytes and macrophages [7]. Furthermore, an imbalance among adiponectin (anti-inflammatory), leptin, and resistin contributes to the development of moderate inflammation associated with obesity and increased adipose tissue cell necrosis. This imbalance underscores the progression of insulin resistance and metabolic syndrome [2, 30]. Our findings indicate that PSEE increases the expression of adiponectin and its receptor, while simultaneously decreasing the expression of leptin and resistin genes. This is the first investigation that determines the properties of PSEE on adiponectin, leptin, and also resistin. These experimental findings offer compelling evidence of the anti-inflammatory properties of PSEE, likely attributed to the presence of compounds like piceatannol and resveratrol. The observed inhibition of proinflammatory cytokines and adipokines associated with inflammation underscores the potential therapeutic significance of passion fruit seed extract in alleviating adipose tissue inflammation.

Inflammation occurring within adipocytes is regulated by the activation of NF-*κ*B and MAPK signaling pathways [17, 19]. In this study, we investigated the impact of PSEE on the expression of ERK1/2, p38MAPK, and NF-*κ*B p65 proteins in cocultured adipocytes and macrophages stimulated with LPS. The results demonstrated a significant reduction in the expression of NF-*κ*B p65 and p38MAPK proteins upon treatment with PSEE. This finding was consistent with a previous study that highlighted the inhibitory effect of resveratrol, a major component of passion fruit seed, on the phosphorylation of ERK1/2 and NF-*κ*B p65 in cocultured adipocytes and macrophages [31]. The anti-inflammatory effects of piceatannol, resveratrol, and *α*-tocopherol are thought to be mediated through the inhibition of the NF-*κ*B pathway activation and/or SIRT1 activation [16]. However, in contrast to the effect on NF-*κ*B and p38 MAPK proteins, PSEE did not show any significant impact on the expression of ERK1/2 proteins in cocultured adipocytes and macrophages stimulated with LPS. These findings suggest that the extract specifically targets the NF-*κ*B and p38 MAPK signaling pathways, while the ERK1/2 pathway remains unaffected. Taken together, the results of this study indicate that the inhibition of NF-*κ*B and p38 MAPK signaling pathways by the PSEE suggests its potential as a therapeutic agent for mitigating adipose tissue inflammation. In addition to the MAPK and NF-*κ*B pathways, the AMP-activated protein kinase (AMPK) and mTOR signaling pathways are intricately linked to the development and regulation of obesity, making them potential targets for therapeutic intervention [14, 32]. The effects of PSEE on the activation of the AMPK and mTOR signaling pathways should be evaluated for further study to understand the underlying mechanisms involved.

Lipolysis in adipocytes constitutes a pivotal metabolic pathway responsible for breaking down triglycerides into fatty acids and glycerol, thus generating energy. Furthermore, these lipolytic processes contribute to the reduction of fat content within adipose tissue [33, 34]. ACC, aP2, FaSN, LPL, HSL, and ATGL are the genes that experience upregulation in mature adipocytes, playing significant roles in the synthesis of triglycerides [35]. In this study, it was observed that PSEE led to an increase in the expression of ACC, HSL, and FaSN genes, while concurrently resulting in a decrease in the expression of aP2, ATGL, and LPL genes in the inflammation-co-cultured adipocytes and macrophages.

## 5. Conclusion

**Figure 6:**
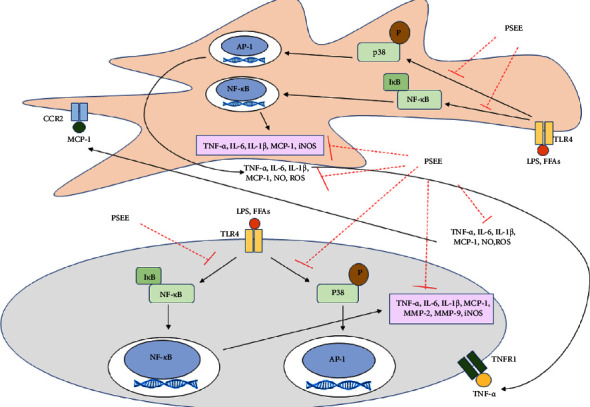
Schematic summary of the inhibitory activity of PSEE in cocultured adipocytes and macrophages.

This study demonstrated that the ethanolic extract of passion fruit seeds effectively reduces ROS and NO levels by inhibiting iNOS protein expression. Additionally, it exerts anti-inflammatory effects in LPS-stimulated adipocytes by suppressing the activation of p38 and NF-*κ*B signaling pathways, resulting in reduced production of proinflammatory cytokines including TNF-*α*, IL-1*β*, MMP-2, IL-6, and MCP-1, as well as decreased adipokine levels. Moreover, the ethanolic extract of passion fruit seeds increased the expression of lipogenic gene, including *ACC, HSL*, and *FaSN*. However, *aP2, ATGL*, and *LPL* were dramatically downregulated (Figure 6). These findings contribute to the understanding of the potential therapeutic applications of passion fruit seed extract in mitigating adipose tissue inflammation and adipogenesis-related disorders. However, further research is necessary to elucidate additional specific mechanisms including AMPK and mTOR signaling pathways. In vivo studies are also required to confirm the effects of passion fruit seed extract against inflammation in an obese animal model.

## Data Availability

The data that support the findings of this study are available on request.
